# Risk factors of lymph node metastasis in the splenic hilum of gastric cancer patients: a meta-analysis

**DOI:** 10.1186/s12957-020-02008-1

**Published:** 2020-09-01

**Authors:** Jun Du, Yangchao Shen, Wenwu Yan, Jinguo Wang

**Affiliations:** Department of Gastrointestinal Surgery, The First Affiliated Hospital, Yijishan Hospital of Wannan Medical College, Wuhu, 241001 China

**Keywords:** Gastric cancer, Splenic hilum lymph node, Risk factors, Meta-analysis

## Abstract

**Background:**

The issue of whether or not splenic hilum lymph nodes (SHLN) should be excised in radical gastrectomy with D2 lymph node dissection remains controversial. In this study, we identified the clinicopathological features in patients with gastric cancer that could serve as predictive risk factors of SHLN metastasis.

**Methods:**

We searched Medline, Embase, PubMed, and Web of Science databases from inception to May 2020 and consulted the related references. Overall, 15 articles evaluating a total of 4377 patients were included for study. The odds ratios (OR) of each risk factor and corresponding 95% confidence intervals (CI) were determined using the Revman 5.3 software.

**Results:**

Our meta-analysis revealed tumor size greater than 5 cm (*p* < 0.01), tumor localization in the greater curvature (*p* < 0.01), diffuse type (Lauren’s classification) (*p* < 0.01), Borrmann types 3–4 (*p* < 0.01), poor differentiation and undifferentiation (*p* < 0.01), depth of invasion T3–T4 (*p* < 0.01), number of lymph node metastases N2–N3 (*p* < 0.01), distant metastasis M1 (*p* < 0.01), TNM stages 3–4 (*p* < 0.01), vascular invasion (*p* = 0.01), and lymphatic invasion (*p* < 0.01) as potential risk factors of SHLN metastasis. Moreover, positivity of Nos. 1, 2, 3, 4sa, 4sb, 4d, 6, 7, 9, 11, and 16 lymph nodes for metastasis was strongly associated with SHLN metastasis.

**Conclusions:**

Tumor size, tumor location, Lauren’s diffuse type, Borrmann type, degree of differentiation, T stage, N stage, M stage, TNM stage, vascular invasion, lymphatic infiltration, and other positive lymph nodes are risk factors for SHLN metastasis.

## Introduction

Despite a downward trend in mortality rates, gastric cancer (GC) remains the third leading cause of cancer-related death and the fifth most commonly diagnosed cancer type worldwide [1]. Surgical resection is currently the only effective means to treat GC. According to the Japanese gastric cancer treatment guidelines, standard gastrectomy involves resection of at least two-thirds of the stomach with D2 lymph node dissection. The No. 10 lymph node is also within the scope of resection in proximal GC [2]. The deep anatomical position of the splenic hilum leads to narrowing of the operative space. Owing to the fragility of the spleen and variability of splenic hilum vessels, SHLNs dissection is difficult to perform [3]. Although splenic hilum lymph nodes can be completely removed with splenectomy, its application remains a subject of debate. An earlier large-scale randomized controlled trial showed no significant differences in the 5-year survival rates between splenectomy and spleen-preserving groups. However, increased morbidity and blood loss were recorded in the splenectomy group [4]. The prognosis of cases with SHLN metastasis is generally poorer than those with no metastasis [5]. The survival benefit of preventive splenic hilum lymphadenectomy remains a controversial issue [3, 6]. In this study, we systematically reviewed the risk factors of SHLN metastasis to evaluate whether spleen-preserving splenic hilum lymph node dissection provides a therapeutic advantage in high-risk patients.

## Materials and methods

### Search strategy

We searched Medline, Embase, Web of Science, and PubMed databases from inception to May 2020 and consulted the reference lists for relevant articles. The following search terms were used: Stomach Neoplasms, Stomach Neoplasm, Gastric Neoplasm, Cancer of Stomach, Stomach Cancer, Gastric Cancer, Lymph Nodes, Lymph Node, No. 10, Splenic Hilar, Splenic Hilum, and Metastasis. A combination of medical subject headings and keywords were used for the search, with no language restrictions.

### Inclusion and exclusion criteria

Inclusion criteria were as follows: (1) patients diagnosed with gastric cancer and subjected to proximal/total gastrectomy with D2/D3 lymphadenectomy, (2) case control studies, (3) literature containing information on risk factors for splenic lymph node metastasis, and (4) Newcastle-Ottawa quality assessment scale (NOS) score greater than 5 points.

Exclusion criteria were as follows: (1) patients diagnosed with residual GC, gastroesophageal junctional cancer, or gastric stromal tumors; (2) overviews, case studies, or abstracts; (3) studies that did not include original data and/or lacked a control group or key information that could not be obtained despite contacting the author; and (4) literature originating from the same institution simultaneously.

### Literature screening, data extraction, and quality evaluation

All the included studies were imported into the Endnote X9 software. After review of the full text, studies were screened according to inclusion and exclusion criteria. We designed an information extraction table, including data on author name, publication date, country, number of cases, age, gender, tumor size, tumor location, Lauren’s classification, Borrmann classification, tumor differentiation, depth of tumor invasion, number of lymph node metastases, distance metastasis, TNM stage, neurological invasion, vascular invasion, lymphatic invasion, and other positive groups of lymph node metastasis. Quality evaluation was performed using NOS [7, 8]. Studies with NOS scores > 7 points were rated as high-quality literature, < 5 points as low-quality literature, and the remaining studies as medium-quality literature. All operations were performed independently by two researchers. In the event of a disagreement, a third researcher resolved the dispute.

### Statistical analysis

Dichotomous variable data were presented as a Forest plot using OR and 95% CI. The Q test and *I*^2^ statistic were used to measure the degree of heterogeneity of the combined data. A random-effects model was used in cases where *I*^2^ > 50% and/or *p* < 0.01; otherwise, a fixed-effects model was used. Sensitivity analysis was conducted by eliminating individual studies and changing the effect model to test the stability of combined data. A funnel plot was employed to evaluate publication bias. Data were considered statistically significant at *p* < 0.05.

## Results

### Study selection

**Fig. 1:**
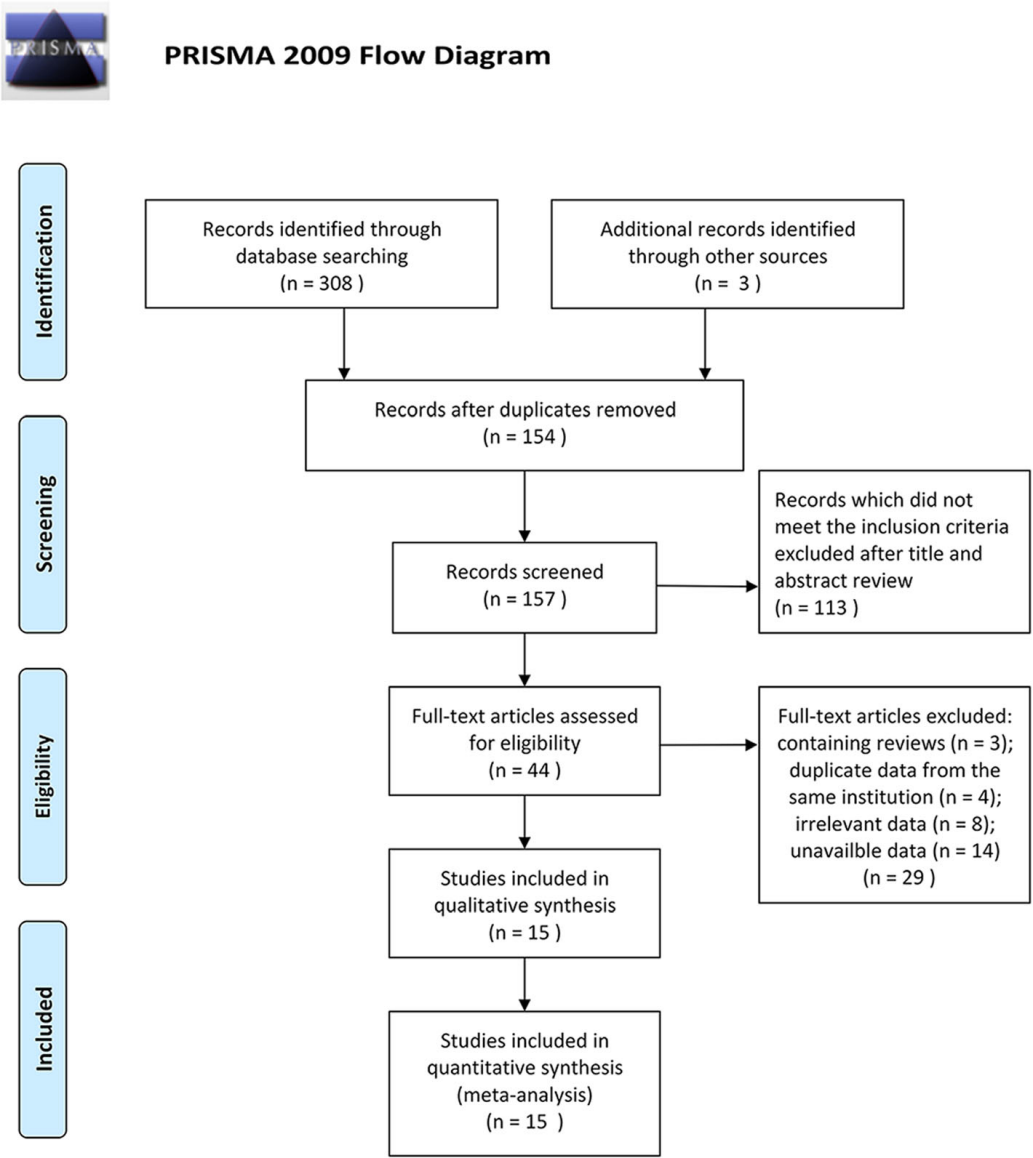
Flow diagram of the search and selection process

We retrieved a total of 308 articles from Medline (*n* = 39), Embase (*n* = 121), PubMed (*n* = 103), and Web of Science (*n* = 45), and obtained three studies from the relevant references. After excluding 154 duplicates, the titles and abstracts of the remaining studies were reviewed, which led to the exclusion of 113 unrelated articles. Following exclusion of review articles and studies analyzing patient data from the same institution over the same time-period as well as irrelevant and unavailable data, 15 articles were finally included (Fig. 1). A total of 4377 patients with GC underwent gastrectomy and lymphadenectomy in the included studies. Seven of the studies were performed in China, five in Japan, two in South Korea, and one in Germany. All studies contained at least one risk factor for SHLN metastasis. The general characteristics and quality assessments of the included studies are listed in Table 1.

**Table 1:** General characteristics of the included studies

Author	Year	Country	Number	Method	Risk factors	Quality
Jeong et al. [9]	2018	Korea	665	Retrospective	2, 4, 5, 6, 8, 9, 10, 14	High/8
Chen et al. [10]	2016	China	243	Retrospective	1, 2, 3, 4, 5, 6, 7, 8, 9, 11, 14	High/8
Bian et al. [11]	2016	China	380	Retrospective	15	High/9
Chen et al. [5]	2014	China	205	Retrospective	1, 2, 3, 4, 5, 6, 8, 9, 11, 14	High/8
Huang et al. [12]	2014	China	346	Retrospective	1, 2, 4, 5, 8, 9, 14, 15	High/9
Zhu et al. [13]	2012	China	265	Retrospective	2, 5, 6, 7, 8, 9, 10	High/8
Zhang et al. [14]	2011	China	590	Retrospective	1, 2, 3, 4, 5, 6, 15	High/8
Kosuga et al. [15]	2011	Japan	280	Retrospective	1, 2, 3, 4, 5, 8	High/8
Aoyagi et al. [16]	2010	Japan	191	Retrospective	1, 2, 3, 4, 5, 6, 8, 9, 12, 13, 14, 15	Moderate/7
Shin et al. [17]	2009	Korea	319	Retrospective	1, 2, 3, 4, 5, 6, 7, 8, 9, 11, 12, 13, 14	Moderate/7
Sasada et al. [18]	2009	Japan	201	Retrospective	2, 8	Moderate/7
Huang et al. [19]	2009	China	237	Retrospective	2, 4, 5, 6, 8, 9	Moderate/6
Kunisaki et al. [20]	2007	Japan	118	Retrospective	2, 3, 4, 5, 8, 12, 13, 14, 15	High/9
Ikeguchi et al. [21]	2004	Japan	225	Retrospective	9	Moderate/6
Stefan et al. [22]	2001	Germany	112	Retrospective	2, 4, 6, 7, 8, 14	Moderate/7

1, age; 2, gender; 3, tumor size; 4, tumor location; 5, depth of invasion; 6, Borrmann type; 7, Lauren’s type; 8, differentiation; 9, lymph node metastasis; 10, distance metastasis; 11, neurological invasion; 12, vascular invasion; 13, lymphatic invasion; 14, TNM stage; 15, positive lymph node metastasis in other groups. High, NOS score > 7 points; low, NOS score < 6 points; moderate, 6–7 points

### Age

**Fig. 2:**
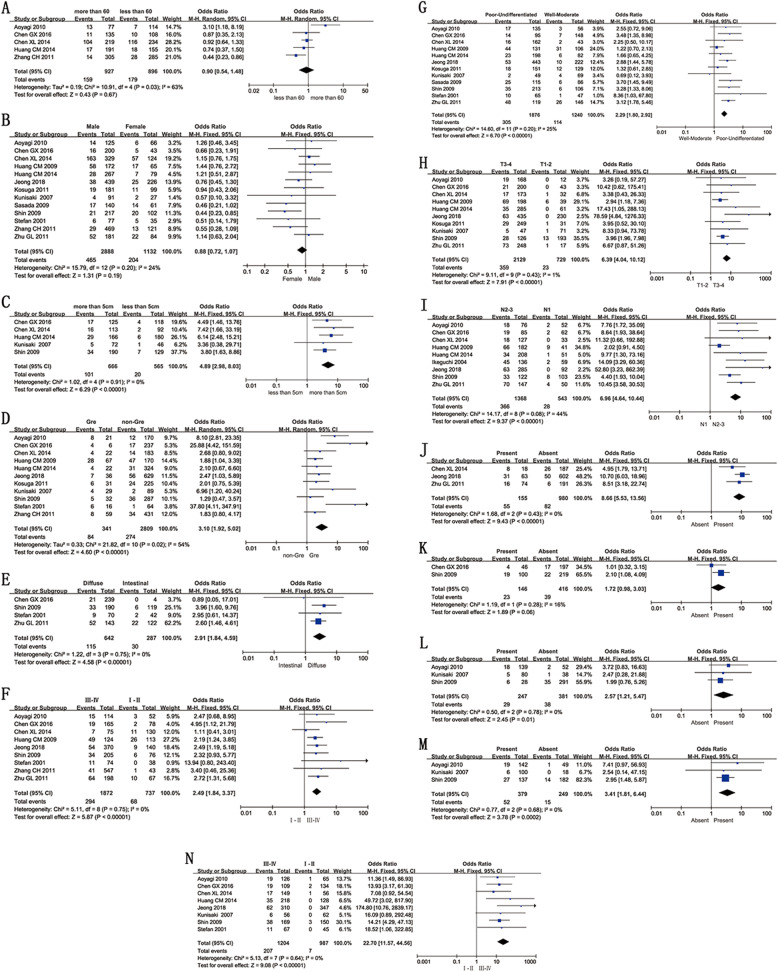
Forest plot analysis of risk factors. **a** Age. **b** Sex. **c** Tumor size. **d** Tumor location. **e** Lauren’s type. **f** Borrmann type. **g** Histological differentiation. **h** Depth of invasion. **i** Lymph node metastases. **j** Distance metastasis. **k** Neurological invasion. **l** Vascular invasion, **m** Lymphatic invasion, **n** TNM stage

Five studies with a total of 1823 GC patients were included for analysis. The number of GC patients with No. 10 LN+ was 159, 179 in patients < 60 years and patients > 60 years, respectively (OR = 0.90, 95% CI = 0.54–1.48, *I*^2^ = 63%, *p* = 0.67). We observed no significant differences between the two groups (Fig. 2a), suggesting that age is not correlated with SHLN metastasis. Owing to study heterogeneity, a sensitivity analysis was conducted by eliminating individual studies. Notably, heterogeneity was significantly decreased when the study of Aoyagi et al. [16] was removed (*I*^2^ = 18%).

### Sex

Thirteen studies (including 2888 males and 1132 females) were used for gender analysis. No significant differences were observed between male and female groups (OR = 0.88, 95% CI = 0.72–1.07, *I*^2^ = 24%, *p* = 0.19; Fig. 2b), indicating that gender is not a risk factor for SHLN metastasis.

### Tumor size

Five studies were included in analysis of tumor size. Due to differences in statistical analyses of tumor diameter, we divided the information into two groups (> 5 cm vs. < 5 cm and > 10 cm vs. < 10 cm). No significant differences were evident between the > 10 cm and < 10 cm groups, and studies had high heterogeneity (OR = 0.58, 95% CI = 0.21–1.58, *I*^2^ = 71%, *p* = 0.28). However, the > 5 cm group exhibited a markedly higher incidence of SHLN metastasis, with no significant study heterogeneity (OR = 4.89, 95% CI = 2.98–8.03, *I*^2^ = 0%, *p* < 0.01; Fig. 2c).

### Tumor location

Eleven studies involving 341 patients with tumors located in the greater curvature (Gre) and 2809 with tumors in other locations were included in the analysis of tumor location. Differences between tumors within the greater curvature and those found elsewhere were significant (OR = 3.10, 95% CI = 1.92–5.02, *I*^2^ = 54%, *p* < 0.01; Fig. 2d). The results suggest that Gre location of tumors is a risk factor for SHLN metastasis. In the sensitivity analysis, heterogeneity did not change significantly upon elimination of individual studies.

### Lauren’s type

Four articles containing 929 patients evaluated Lauren’s type, which included both diffuse and intestinal subtypes. After combining the data, significant differences were evident between diffuse and intestinal types (OR = 2.91, 95% CI = 1.84–4.59, *I*^2^ = 0%, *p* < 0.01; Fig. 2e), indicating that diffuse type is associated with an increased incidence of SHLN metastasis.

### Borrmann type

Nine articles included information regarding Borrmann type. Based on the Borrmann classification system, GCs are divided into four types (I, II, III, and IV). We combined types I–III and compared the data with type IV. The overall heterogeneity of the two groups was small, and type IV was associated with a significantly increased risk of SHLN metastasis (OR = 2.49, 95% CI = 1.84–3.37, *I*^2^ = 0%, *p* < 0.01; Fig. 2f).

### Histological differentiation

Twelve studies provided data on histological differentiation. We set poorly differentiated and undifferentiated types as the exposure groups and moderately differentiated and well-differentiated types as the control groups. Subsequently, studies were individually removed for sensitivity analysis. Our results showed no significant changes in heterogeneity, and the poorly differentiated and undifferentiated types were associated with significantly higher risk of SHLN metastasis (OR = 2.29, 95% CI = 1.80–2.92, *I*^2^ = 25%, *p* < 0.01; Fig. 2g).

### Depth of invasion

Data regarding depth of invasion were included in 10 studies. T3 and T4 were set as the exposure group and T1 and T2 as the control group. There was no significant heterogeneity in either group, and T3 and T4 groups exhibited a markedly higher rate of SHLN metastasis than T1 and T2 groups (OR = 6.39, 95% CI = 4.04–10.12, *I*^2^ = 1%, *p* < 0.01; Fig. 2h).

### Lymph node metastasis

Nine studies provided information on lymph node metastases, including N1, N2, and N3. N1 and N2–3 were grouped separately. After the data were combined, GC patients with N2 or N3 exhibited a significantly increased risk of SHLN metastasis (OR = 6.96, 95% CI = 4.64–10.44, *I*^2^ = 44%, *p* < 0.01; Fig. 2i). Owing to heterogeneity, we conducted a sensitivity analysis by eliminating individual studies and found that heterogeneity disappeared when the study of Huang et al. [19] was removed (*I*^2^ = 0%).

### Distant metastasis

Three articles, including 1272 patients, provided data on distant metastases. After the data were combined, no heterogeneity was evident, and distant metastasis was associated with a significantly higher rate of SHLN metastasis (OR = 8.66, 95% CI = 5.53–13.56, *I*^2^ = 0%, *p* < 0.01; Fig. 2j).

### Neurological, vascular, and lymphatic invasion

We excluded several studies reporting combined data on blood vessels and lymphatics. Two studies included data on neurological invasion. Upon combination of data from both studies, no significant differences between the SHLN+ group and the SHLN− group were evident (OR = 1.72, 95% CI = 0.98–3.03, *I*^2^ = 16%, *p* = 0.06; Fig. 2k). Three studies examined vascular invasion. When the data were combined, differences between the SHLN+ group and the SHLN− group were significant (OR = 2.57, 95% CI = 1.21–5.47, *I*^2^ = 0%, *p* = 0.01; Fig. 2l). Three articles provided information on lymphatic invasion. Upon combination of the data, we observed a significant difference between the two groups (OR = 3.41, 95% CI = 1.81–6.44, *I*^2^ = 0%, *p* < 0.01; Fig. 2m). Data from our meta-analysis collectively suggest that the presence of vascular and lymphatic invasion is a significant risk factor for SHLN metastasis.

### TNM stage

Eight articles reported TNM stage that was classified into four subtypes (I, II, III, and IV). We combined types I–III into a single group for comparison with type IV. The heterogeneity of the two groups was small, and type IV was associated with a significantly increased risk of SHLN metastasis (OR = 22.70, 95% CI = 11.57–44.56, *I*^2^ = 0%, *p* < 0.01; Fig. 2n).

### Other groups with positive lymph node metastasis

Four studies referred to other regional lymph node metastases, which were associated with SHLN metastasis. Their combined values and effects are listed in Table 2. The results showed that other regional lymph nodes, with the exception of No. 5 LN (*p* = 0.14) and No. 8a LN (*p* = 0.10), are associated with SHLN metastasis. Sensitivity analysis conducted by changing the effect model revealed no significant heterogeneity.

**Table 2:** Other groups with positive lymph node metastasis

Regional LN stations (+)	Studies	Effect model	Pooled OR	95% CI	***I***^**2**^	***p*** value
No. 1	3	Fixed	1.76	1.19–2.60	0%	< 0.01
No. 2	4	Random	2.38	1.06–5.32	80%	0.04
No. 3	4	Random	3.65	1.74–7.67	58%	< 0.01
No. 4sa	2	Fixed	17.71	10.35–30.30	0%	< 0.01
No. 4sb	3	Fixed	6.91	4.50–10.62	0%	< 0.01
No. 4d	2	Fixed	4.54	2.32–8.87	0%	< 0.01
No. 5	3	Fixed	1.77	0.90–3.46	0%	0.14
No. 6	3	Fixed	1.74	1.03–2.94	31%	0.04
No. 7	4	Random	3.05	1.62–5.73	64%	< 0.01
No. 8a	2	Fixed	1.61	0.92–2.81	0%	0.10
No. 9	4	Random	2.83	1.22–6.56	76%	0.02
No. 11	4	Fixed	3.92	2.81–5.49	47%	< 0.01
No. 16	2	Fixed	4.34	1.08–17.39	44%	0.04

### Publication bias

**Fig. 3:**
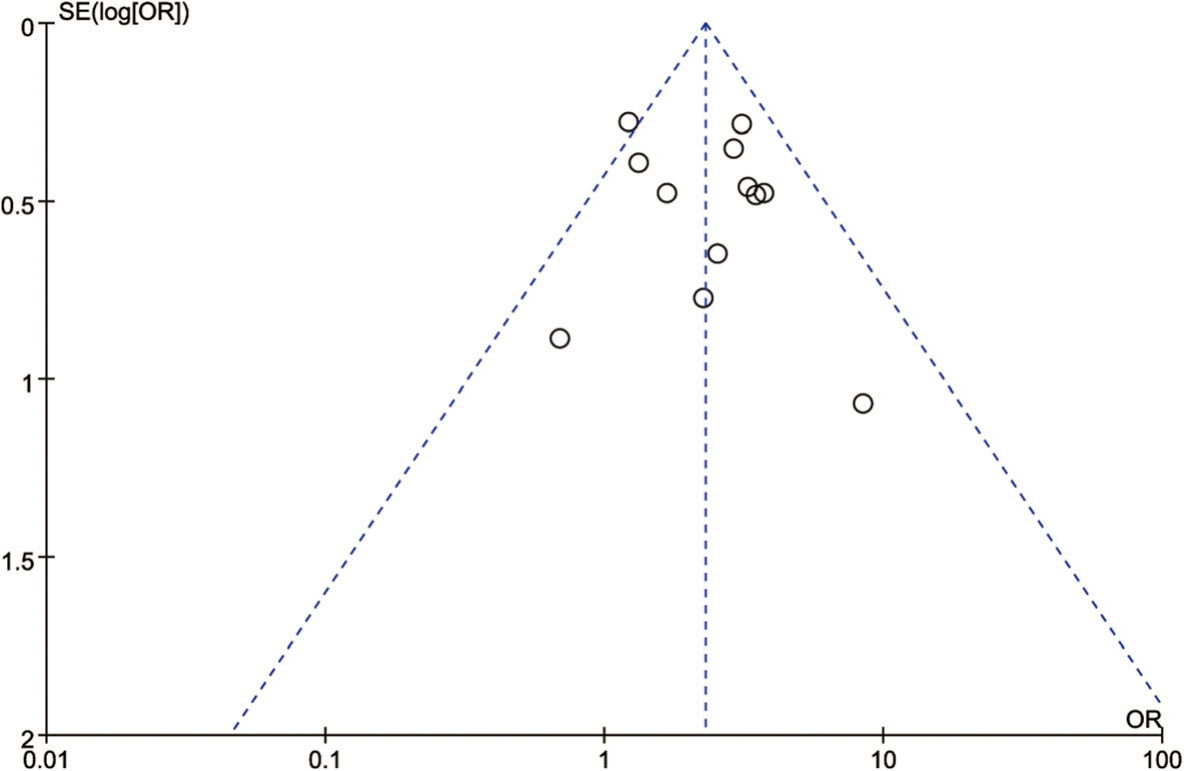
Funnel plot of histological differentiation

Publication bias was assessed only when more than 10 studies were included in the risk factor analysis. We observed no obvious asymmetry in the Funnel plot of histological differentiation (Fig. 3). Similarly, other aggregated data did not exhibit publication bias.

## Discussion

While the mortality rate of gastric cancer has decreased over recent years, metastasis to splenic hilum lymph nodes is associated with poor prognosis [23]. To date, risk factors for SHLN metastasis have been assessed, but not the impact of other regional lymph nodes. In this study, we identified 11 potential risk factors for SHLN metastasis. Among the clinicopathological features, T stage (OR = 6.39), N stage (OR = 6.96), M stage (OR = 8.66), and TNM stage (OR = 22.70) were strongly associated with No. 10 lymph node metastasis. Lymph from the stomach wall flows into the submucosal lymphatic network and eventually into the peri-gastric lymphatic system [24]. Therefore, greater tumor depth facilitates invasion of lymphatic vessels, leading to a high rate of lymph node metastasis. Jeong et al. reported that all patients displaying SHLN metastasis were diagnosed with stage 3 or 4 disease, while patients with no lymph node metastasis of splenic hilum were stage 1 or 2 [9].

Generally, cancer located in the upper part or greater curvature of the stomach tends to metastasize to the splenic hilum, which may be associated with the lymphatic reflux pathway in the region [25–27]. Gastric adenocarcinoma located in the upper one-third of the greater curvature is drained to SHLNs through lymphatic vessels of the posterior gastric artery (4sa) [24, 28]. In an earlier study, Takahashi et al. injected activated carbon particles under serosa and showed that when the tumor is located in the middle one-third of the greater curvature of the stomach, lymph flows to the upper or lower part of the peri-gastric lymph nodes (4sb, 4d) [29]. Experiments by Yura et al*.* showed that the rates of lymph node metastasis of Nos. 4sa, 4sb, 4d, and 10 were significantly higher than those of tumors located in the non-greater curvature side [27], consistent with our finding that Nos. 4sa (OR = 17.71), 4sb (OR = 6.91), and 4d (OR = 4.54) LN metastases are strongly associated with No. 10 LN metastasis. In the normal lymphatic reflux pathway, metastasis to some higher lymph node stations, such as Nos. 11 (OR = 3.92) and 16 (OR = 4.34), may also be indicative of SHLN metastasis. However, no significant differences between No. 16-positive and No. 10-positive cases have been reported to date [30, 31].

In view of the complex anatomical location of the splenic hilum, the issues of whether SHLNs should be dissected and splenectomy or spleen-preserving lymph node dissection performed are under debate [6, 32–35]. According to the Japanese guidelines for the treatment of gastric cancer, total gastrectomy and D2 lymphadenectomy should be advocated for advanced gastric cancer in the upper region. However, an earlier report showed a poorer overall survival rate of patients subjected to lymph node dissection relative to the non-dissected group when SHLN metastasis occurred. The majority of cases in this study were advanced GC, which could lead to risk of bias [3]. A randomized controlled trial showed no significant differences in 5-year survival rates between patients receiving total gastrectomy and those receiving total gastrectomy with splenectomy [4]. Thus, the survival benefit of lymph node dissection for proximal GC remains controversial. In the past, splenectomy has been considered to allow for complete resection of lymph nodes in the splenic hilum. However, subsequent experiments have demonstrated no significant differences in the 5-year survival rates between splenectomy and non-splenectomy groups [6, 33]. A number of reports in the literature suggest that splenectomy has limited benefits [34, 35]. For instance, a recent large-scale randomized controlled trial in Japan demonstrated that splenectomy resulted in greater morbidity and complications and did not increase the survival benefits of patients. However, GC patients with simple splenic hilum lymph node metastasis are relatively rare, resulting in a lack of relevant randomized controlled trials. Therefore, the issue of whether surgical removal of the spleen provides survival benefits remains to be established [4].

In terms of surgical treatment, adoption of reasonable and individualized strategies considering safety and quality of life is a new trend in Japan [36]. It is becoming increasingly necessary to implement individualized treatments to ensure optimal patient outcomes. In cases where tumors are at an advanced stage or located in either the greater curvature of the stomach with Nos. 4sa, 4sb, or 4d lymph node metastasis or lesser curvature of the stomach with Nos. 3, 7, or 11 lymph node metastasis, splenic hilum lymph node dissection is recommended. Spleen-preserving splenic hilum lymph node dissection may be considered in the presence of other risk factors.

Our study has a number of limitations that should be acknowledged. (1) All the included studies were retrospective and limited by language and region, which could increase the risk of bias. (2) There were significant heterogeneities in a number of parameters between some studies, which may be related to factors such as surgical operations, pathological diagnosis, and statistical analysis. (3) In some cases, complete data could not be obtained, potentially resulting in deviation of results. (4) *p* values of around 0.05 were obtained for some of the analyses, such as neurological invasion and Nos. 2, 6, and 16 lymph node metastasis. Our preliminary conclusions based on these results should therefore be treated with caution.

## Conclusions

Based on the results of our meta-analysis, 11 risk factors of SHLN metastasis in GC patients were ultimately identified. According to OR data, tumor size, tumor location, Lauren’s type, Borrmann type, histological differentiation, depth of invasion, lymph node metastases, distant metastasis, vessel invasion, TNM stage, and Nos. 1, 2, 3, 4sa, 4sb, 4d, 6, 7, 9, 11, and 16 positive lymph nodes were strongly associated with SHLN metastasis. Knowledge of the associations of these clinicopathological features with SHLN metastasis can help clinicians assess patients and develop individualized surgical plans. However, the main drawbacks of the current study are the limited number of included studies, mainly located in Asian countries, and lack of detailed data. Therefore, study of risk factors of SHLN metastasis should be combined with data from other groups of lymph node metastasis and further verified with the aid of multi-center and large-scale prospective analyses in the future.

## Data Availability

All the data analyzed were obtained from the original articles.

## Abbreviations

SHLN: Splenic hilum lymph node
GC: Gastric cancer
NOS: Newcastle-Ottawa quality assessment scale
